# Diagnosing and Treating Preseptal Cellulitis in Pediatric Patients After a Minor Trauma

**DOI:** 10.7759/cureus.74211

**Published:** 2024-11-22

**Authors:** Alara Kiliccioglu, Ashraf Rakhimov, Hasan Aldinc

**Affiliations:** 1 Ophthalmology, Acibadem University, Istanbul, TUR; 2 Pediatric Emergency Medicine, Kanuni Sultan Süleyman Training and Research Hospital, Istanbul, TUR; 3 Emergency Medicine, Acibadem University Hospital Atakent, Istanbul, TUR

**Keywords:** eye infection, eyelid hygiene, ocular complications, preseptal orbital cellulitis, trauma pediatric

## Abstract

Preseptal cellulitis is a commonly observed inflammation of the eyelid and the surrounding skin in pediatric patients, especially after a minor trauma. Although preseptal cellulitis is generally associated with a more favorable prognosis, it is vital to remember that all orbital infections require prompt diagnosis and treatment because of the risk of severe complications. Inadequate or failure to adhere to the treatment plan and unmet hygiene standards can lead to severe complications; therefore, diligent follow-up care should be undertaken by both the physician and the patient.

## Introduction

Preseptal cellulitis is a common infection in the anterior of the orbital septum involving the superficial and soft tissue of the palpebra and periorbital region. It manifests with edema, hyperemia, and pain in the palpebral and periorbital tissue [1]. As preseptal cellulitis is limited to the anterior of the orbital septum, it is generally associated with a more favorable prognosis; however, prompt diagnosis and treatment are crucial for all orbital infections. Failure to adhere to treatment modalities can lead to a series of complications, such as orbital cellulitis [2]. In cases of orbital cellulitis, unlike preseptal cellulitis, involvement of the eyeball and surrounding tissues (eye muscles, adipose tissue, etc.) is seen, and it usually presents with pain upon eye movement [3]. Left untreated, orbital cellulitis can lead to significant complications, including vision loss, meningitis, and intracranial abscesses [4]. Hence, diligent follow-up care and adherence to treatment are necessary to ensure favorable outcomes and minimize the risk of complications.

In this case report, we review the treatment options for preseptal cellulitis in pediatric patients, particularly after a minor trauma. Although preseptal cellulitis is much more common and a mild condition, sometimes it can be difficult to distinguish it from orbital cellulitis, since erythema, swelling, and ocular pain are common initial findings of both conditions [5]. Therefore, an initial exam should be carried out thoroughly for an accurate diagnosis. Early intervention, such as the prompt removal of debris around lacerations and irrigation with balanced saline solutions, should be undertaken. Diligent follow-ups, appropriate selection of antibiotics effective against both gram-negative and gram-positive bacteria, adherence to treatments, and patient education are crucial for stopping disease progression and preventing potential complications.

## Case presentation

A nine-year-old boy was admitted to the emergency department of Acibadem University Hospital Atakent on January 14, 2024, on Sunday, after a bicycle accident. The patient, who was wearing his prescription glasses for previously diagnosed myopia and astigmatism, presented with a laceration beneath his right brow bone. He did not claim to suffer from a headache, either before or after the fall, and denied other complaints such as blurred vision and/or vision loss, syncope, nausea, or vomiting. 

Detailed history did not reveal any pathologies regarding ophthalmologic or neurologic diseases.

**Figure 1:**
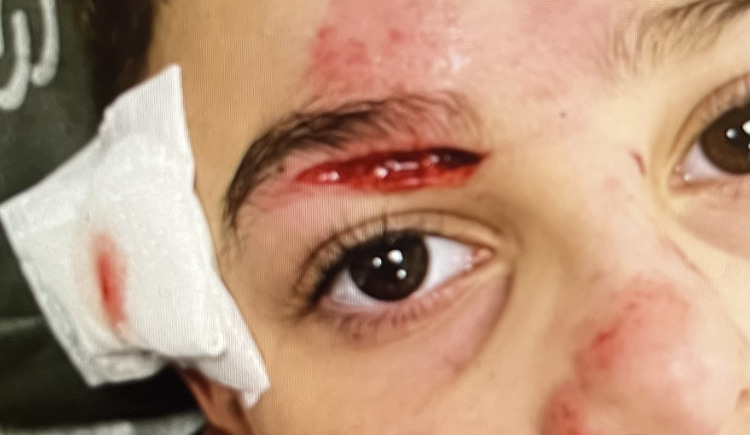
Initial examination, January 14, 2024 Upon initial examination, a 1-cm-deep, 5-cm-long horizontal laceration from the right brow bone to the innermost corner of the right eye was recorded

Upon initial examination, a 1-cm-deep, 5-cm-long horizontal laceration from the right brow bone to the innermost corner of the right eye was recorded (Figure 1). Extraocular movements were intact, pupillary light reflexes were normal, pupils were isochoric, and no nystagmus was seen. The patient was responsive and cooperative and had full orientation, with a blood pressure (BP) of 110/69 mmHg, a pulse rate of 78/m, and a 36.5°C body temperature.

For further testing, a lateral nasal bone graphy was the imaging study preferred due to nasal root sensitivity, and brain CT was opted out from the imaging studies due to the lack of neurological indications and upon the family's wishes. Both the patient and the family were advised to return to the emergency room (ER) for further evaluation, in case there was a change in the patient's condition before they were discharged. Upon their wishes, they were discharged before acquiring stitches to the wound, as they preferred to get them from another healthcare center.

The patient was readmitted on January 17, 2024, on Wednesday, with complaints of increased body temperature, significant swelling around the right eye, and hyperemia.

History regarding the past couple of days showed that after acquiring stitches from a nearby hospital on the 14th, the patient was prescribed oral antibiotics. 

Although amoxicillin/clavulanic acid PO was prescribed to be started on the following day, the patient did not adhere to the prescribed antibiotic regimen, which was perceived as unmanageable by the child; hence, the symptoms were aggravated.

Another private ophthalmology clinic was visited on January 16, 2024. Upon examination, the wound appeared to be mildly hyperemic; hence, topical antibiotic ointments (neomycin and bacitracin, twice a day), as well as eye drops (sodium hyaluronate, four times a day, limited to one day use) and a single-dose eye drop (netilmicin), were prescribed. 

**Figure 2:**
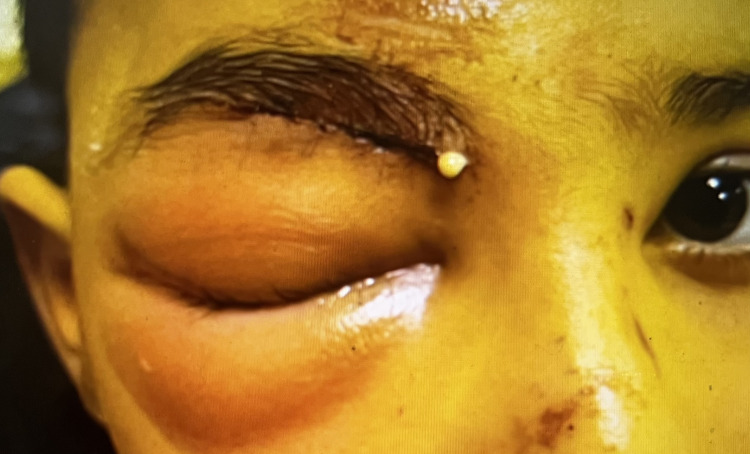
Preseptal cellulitis of the right eye, January 17, 2024 Significant swelling and hyperemia on the right periorbital area with purulent discharge

Examination on January 17, 2024, in our hospital showed significant swelling and hyperemia on the right periorbital area. The area was tender to touch, and a mild fever was present, with a body temperature of 38.3°C. Around the suture lines, foul-smelling purulent drainage was present (Figure 2).

Ocular motility was intact with no restrictions, no nystagmus was seen with isochoric and reactive pupils, and no ophthalmoplegia was recorded. 

In the ophthalmologist consultation, the examination yielded no additional pathologies.

Lab results showed no increase in white blood cell count 8.20×10^3^/uL (normal range: 4-13×10^3^/uL), whereas an increase in C-reactive protein of 5.53 mg/dL (normal range: <0.79 mg/L) was highlighted. 

Regarding the microbiological investigations, the swab taken from the suture line came back as *Staphylococcus aureus* (*S. aureus*) and methicillin-resistant *Staphylococcus aureus* (MRSA) positive.

CT scan of the orbit and sinuses presented diffuse thickening and an increase in density in surrounding subclinical fatty planes, which were interpreted as signs of inflammation and edema. These findings supported the preliminary preseptal diagnosis.

Initial management with intravenous (IV) therapy was deemed necessary considering the patient's history. On January 17, 2024, the patient was admitted to the pediatric wards, and IV ceftriaxone, 2×1650 mg, and teicoplanin, 1×400 mg, were initiated with the diagnosis of preseptal cellulitis. Upon consultation with pediatric infectious diseases on January 18, 2024, the antibiotics previously mentioned were planned to be administered for a minimum of one week (until January 24, 2024), and cefuroxime, a second-generation cephalosporin, 1×1 pomade was added to the treatment regimen. Administration of clindamycin, 4×300 mg, was suggested, for a duration of a day.

**Figure 3:**
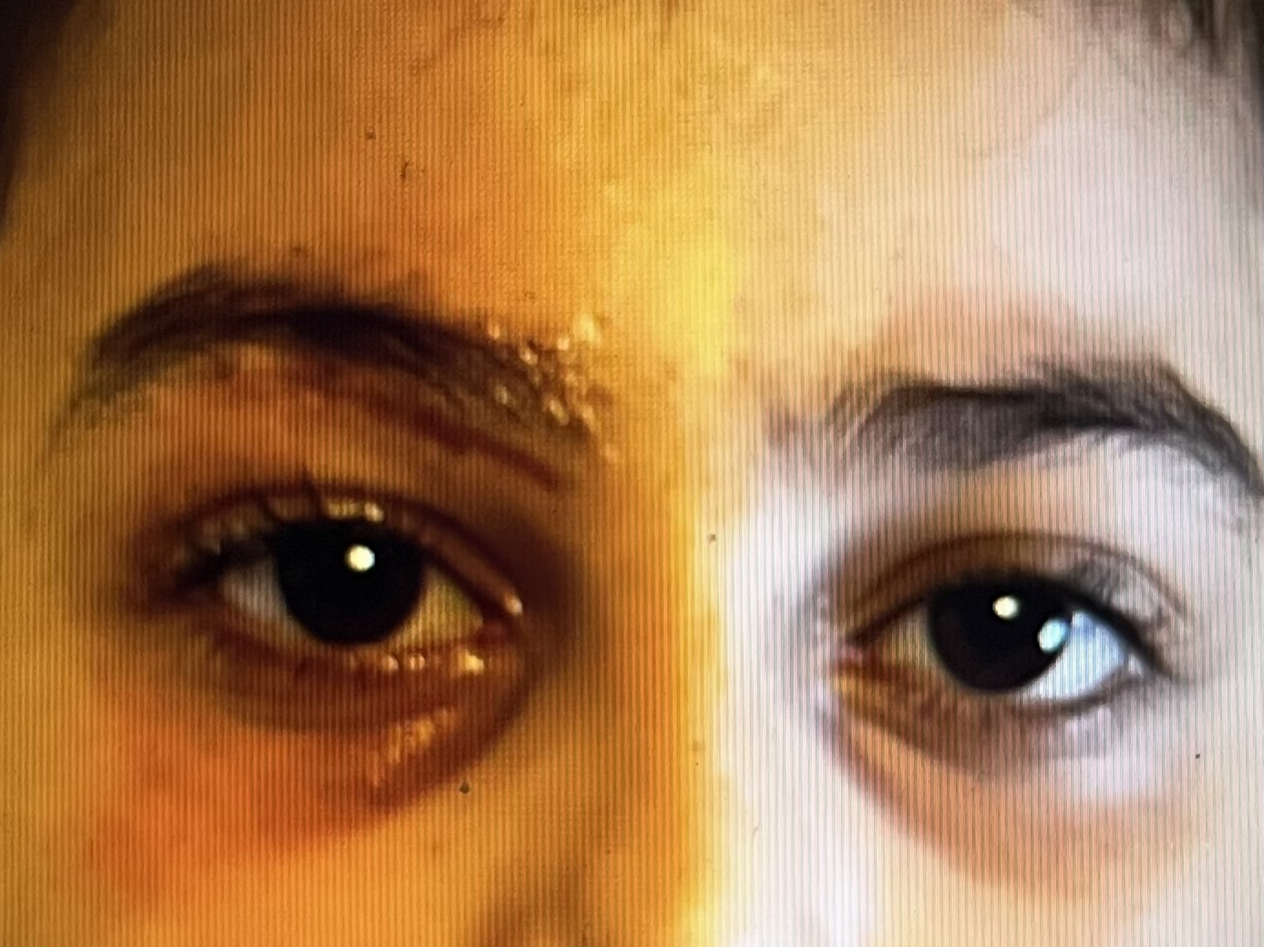
Last examination before discharge, January 24, 2024 Significant improvement was recorded after intravenous treatment and diligent wound care. Apart from the scar under the right brow bone, side by side shows no apparent swelling or discoloration between the right and left eyes

In the following days, the edema and erythema findings regressed significantly with no fever spikes recorded. The hyperemia and edema continued to regress, there was no ophthalmoplegia, and extraocular movements were intact with no restrictions in all planes. Upon finishing the treatment and with the clinical outcome improving, the patient was discharged with no other instructions (Figure 3). 

## Discussion

Preseptal cellulitis describes an infection of the eyelid and superficial periorbital soft tissues without the involvement of the globe and orbit [6]. However, a complete ocular examination of an acutely ill child with tender and swollen eyelids could be hard, and an accurate diagnosis on a clinical basis can be difficult [7]. Therefore, if orbital cellulitis is suspected or if bedside examination cannot rule it out, a sinus CT with contrast is warranted within 24 hours of presentation [8,9].

In cases of mild preseptal cellulitis in adults and children older than one year of age, treatment is typically rendered on an outpatient basis with empiric broad-spectrum oral antibiotics, but in cases where patients fail to respond or demonstrate clinical worsening, they should be promptly transitioned to IV antibiotics [3,10]. Buchanan et al. [11] state that if there is suspicion of periorbital or orbital cellulitis, children should be referred rapidly for comprehensive pediatric assessment and management, with further input from ophthalmology and, in some cases, ear, nose, and throat (ENT) specialists. An urgent CT scan should be carried out if orbital cellulitis or its associated complications are suspected [10]. Overall, there seems to be a consensus that both preseptal and postseptal orbital cellulitis in children may be first managed conservatively with antibiotics [12]. It is also stated that most pediatric patients require admission and IV antibiotics should be started. Once clinical improvement is noted, the patient can be switched to oral antibiotics [13]. Regression of the eyelid edema and erythema, absence of proptosis or swelling, pupil with normal reaction to light, normal conjunctiva, and normal ocular movements are indicators for the improvement of the patient's clinical status. It is vital to remember that clinical improvement should be seen within 24-48 hours and, if no improvement is noted, prompt reassessment and adjustment of the treatment shall be carried out by medical professionals.

In addition to treatment modalities, wound care in trauma patients holds an important place in the prognosis. Follow-up care should be carried out diligently, and if clinical improvement is not observed in the following days, hospitalization for IV treatment should be carried out promptly. 

## Conclusions

Preseptal cellulitis is typically managed on an outpatient basis when no complications are present and treatment protocols and hygiene standards are strictly adhered to. However, failure to comply with these treatment modalities can lead to an increased risk of complications. In such cases, it is essential to reassess the patient and, if deemed necessary, to admit the patient for close observation. This approach is crucial in preventing disease progression and mitigating the potential for serious complications.
